# Single-Cell Electroporation with Real-Time Impedance Assessment Using a Constriction Microchannel

**DOI:** 10.3390/mi11090856

**Published:** 2020-09-16

**Authors:** Yifei Ye, Xiaofeng Luan, Lingqian Zhang, Wenjie Zhao, Jie Cheng, Mingxiao Li, Yang Zhao, Chengjun Huang

**Affiliations:** 1 R&D Center of Healthcare Electronics, Institute of Microelectronics, Chinese Academy of Sciences, Beijing 100029, China; yeyifei@ime.ac.cn (Y.Y.); luanxiaofeng@ime.ac.cn (X.L.); zhanglingqian@ime.ac.cn (L.Z.); zhaowenjie@ime.ac.cn (W.Z.); chengjie@ime.ac.cn (J.C.); limingxiao@ime.ac.cn (M.L.); 2 School of Future Technology, University of Chinese Academy of Sciences, Beijing 100049, China

**Keywords:** microfluidics, intracellular cargo delivery, single-cell electroporation, electroporation effect assessment

## Abstract

The electroporation system can serve as a tool for the intracellular delivery of foreign cargos. However, this technique is presently limited by the inaccurate electric field applied to the single cells and lack of a real-time electroporation metrics subsystem. Here, we reported a microfluidic system for precise and rapid single-cell electroporation and simultaneous impedance monitoring in a constriction microchannel. When single cells (A549) were continuously passing through the constriction microchannel, a localized high electric field was applied on the cell membrane, which resulted in highly efficient (up to 96.6%) electroporation. During a single cell entering the constriction channel, an abrupt impedance drop was noticed and demonstrated to be correlated with the occurrence of electroporation. Besides, while the cell was moving in the constriction channel, the stabilized impedance showed the capability to quantify the electroporation extent. The correspondence of the impedance variation and electroporation was validated by the intracellular delivery of the fluorescence indicator (propidium iodide). Based on the obtained results, this system is capable of precise control of electroporation and real-time, label-free impedance assessment, providing a potential tool for intracellular delivery and other biomedical applications.

## 1. Introduction

Intracellular delivery of foreign cargos is becoming a critical step in biomedical applications, ranging from gene editing to cell-based therapies [1]. In general, the intracellular delivery methods could be divided into the carrier-mediated methods and membrane-disruption-based methods [2,3]. Biochemical methods always need specifically designed carriers loading materials for specific cells, suffering from complexity, toxicity and limited delivery efficiency [4,5]. On the contrary, the physical methods based on membrane disruption have the advantage of the cell/material independency for various applications [6,7,8,9,10,11]. As one of the widely used physical methods, electroporation has gained a lot of attention due to its ease of operation and high delivery efficiency [6,12]. Electroporation is a transient increase in the cell membrane permeability when the cell is exposed to a sufficient electric field, thus allowing intracellular delivery of impermeable foreign cargos [13,14]. In a conventional bulk electroporation (BEP) system, a mixture of cells and cargos is usually suspended in a cuvette full of conducting buffer, and two macro-electrodes with high voltage (~hundreds of volts) are applied [15]. These systems suffer from low delivery efficiency and intense cell damage due to the inhomogeneous, uncontrollable electric field across the cell suspension.

Micro/nano-electroporation (MEP/NEP) systems have been proposed with the advantage of a more intensive electric field with lower applied voltage than conventional BEP systems [11,15,16,17,18,19,20,21]. More importantly, when the dimension of the microfabricated devices is similar to the size of a single cell, electroporation at the single-cell level could be accomplished [18,22,23,24,25]. Compared to analyzing millions of cells in BEP systems, the single-cell electroporation systems could not only enable the precise control of the intracellular delivery, but also provide detailed information of electroporation mechanisms. Khine et al. proposed a constriction microchannel (4 × 3.1 μm) for single-cell trapping and electroporation, with optical observation and electrical measurement [26]. Boukany et al. utilized a micro-nano-micro-channel for the precise delivery of transfection agents to the trapped single cells [27]. In single-cell electroporation systems, complicated micro/nano-fluidic channels [26,27,28,29] or single-cell micromanipulation units [30,31,32,33] were usually required. In addition, most of these systems were combined with an optical detection tool for visualizing electroporation results [34]. The impermeable nuclear dyes (e.g., propidium iodide, PI), fluorescent proteins or dextran as indicators were most commonly used to observe the accumulation of fluorescence inside cells. Although the optical detections have a high spatial resolution, the time resolution is limited by the sensitivity of the optical systems as well as the size of molecules [35]. As a sufficient number of fluorescent molecules accumulated inside cells result in the detectable fluorescence, the optical signals always lag behind the occurrence of electroporation. Besides, the use of fluorescent dyes is unsuitable for subsequent assays of the treated cells. Recently, electrical detections of the current [26,36] and impedance [30,37,38,39,40,41] have been reported to be incorporated with the electroporation system. Compared to the traditional optical detections, electrical detections provide a label-free and instant strategy for electroporation assessment. 

**Figure 1:**
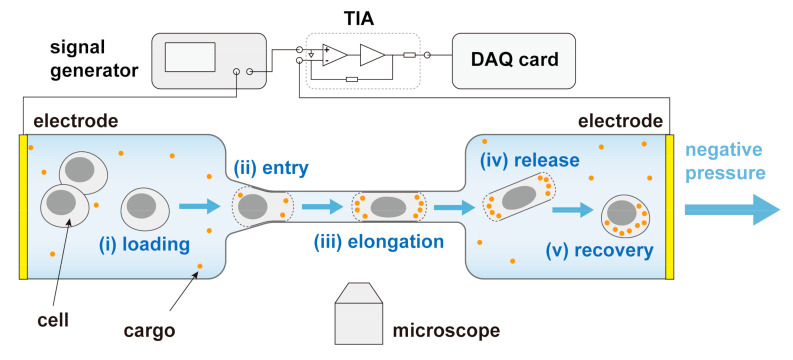
Schematic of the microfluidic system for single-cell electroporation and impedance assessment. This system consists of a microfluidic chip containing a constriction microchannel, a pair of Ag microelectrodes, a signal generator, a trans-impedance amplifier (TIA), a data acquisition (DAQ) card, a microscope, as well as a pressure calibrator.

In this study, we propose a microfluidic system (shown in Figure 1) for single-cell electroporation and real-time impedance monitoring without sacrificing the throughput. The core component of the system is a microfluidic chip with a specially designed constriction channel. Single cells could be aspirated through the constriction channel where the electric field was concentrated, thus ensuring continuous and rapid electroporation. Due to the local high intensity of the electric field focused in the constriction channel, highly efficient electroporation could be achieved with low applied voltage. In addition, the simultaneous impedance variation during cell passing could be utilized to capture the electroporation moment and reveal the electroporation extent for single cells. Compared to cell-trapped-based methods [26,36], this flow-through system not only enables accurate and label-free monitoring of single-cell electroporation, but also shows greatly improved throughput for the demand of rapid intracellular delivery applications.

## 2. Materials and Methods 

### 2.1. Finite Element Method (FEM) Simulation

**Figure 2:**
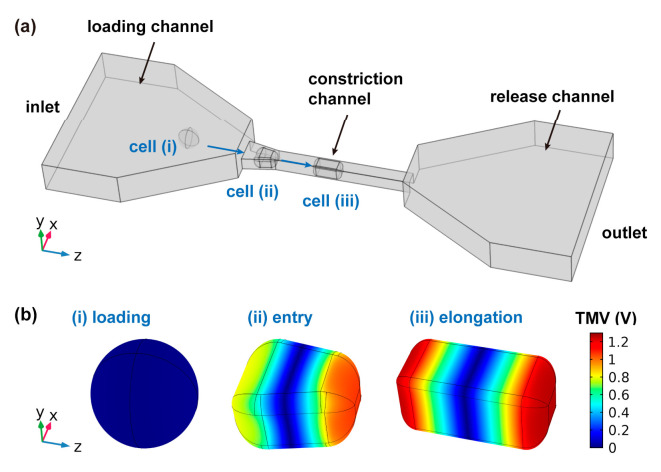
(**a**) Simulation model of the microfluidic channel with a single cell in three locations. (**b**) Simulations of the transmembrane voltages (TMV) of the single cell in the loading channel (i), the entry of the constriction channel (ii) and the center of the constriction channel (iii) respectively, with a voltage of 3 V.

For cell electroporation, the transmembrane voltage (TMV) is a crucial parameter, which can be induced by the external electric field [13,42]. As the efficient charging of TMV could result in the occurrence of electroporation, the FEM simulations of TMVs during cell-passing through the constriction channel were analyzed in COMSOL Multiphysics (COMSOL Multiphysics, COMSOL Inc., Burlington, MA, USA). A three-dimensional (3D) model, including the microchannel and a single cell was established, as shown in Figure 2a. The microchannel consisted of a loading channel, a constriction channel and a release channel. The heights of the loading channel and the release channel were 25 μm. Moreover, both the height and width of the constriction channel were designed as 10 μm to ensure single-cell compression. A single cell was modeled as the cytoplasm domain surrounded by a thin layer of the membrane (thickness of 10 nm). To simulate the cell TMV variation during the cell-entering process, a single cell was simulated at three different statuses: cell in the loading channel, cell in the entry of the constriction channel and cell in the center of the constriction channel, respectively. The parameters in our simulation are shown in Table 1. The electric potential of 3 V was applied to the inlet boundary, and the outlet boundary was grounded. Moreover, other boundaries were electrically insulated. Tetrahedral meshes were built in our simulations, which consisted of 1,270,696, 2,118,736 and 2,353,960 domain elements when the cell was in the loading channel, in the entry of the constriction channel and in the center of the constriction channel, respectively.

### 2.2. Cell Sample Preparation

The lung cancer cell line of A549 was purchased from China Infrastructure of Cell Line Resources (Beijing, China) and cultured with RPMI-1640 media (Gibco, Carlsbad, CA, USA), supplemented with 10% fetal bovine serum (Gibco, Carlsbad, CA, USA) and 1% penicillin/streptomycin (Gibco, Carlsbad, CA, USA) in a cell incubator (3111, Thermo Fisher Scientific, Waltham, MA, USA) at 37 °C in 5% CO_2_. Before experiments, cells in logarithmic phases were trypsinized, and pelleted via centrifugation. Then, three million (3 × 10^6^) cells were resuspended in 1 mL cell culture medium and prepared for the experiments.

### 2.3. Microfluidic Device Design and Fabrication

The microfluidic chip contains a loading channel, a bypass channel, a constriction channel and a release channel, as shown in Supplementary Figure S1. In order to avoid cell clogging around the inlet of the constriction channel, a bypass channel was designed to be connected with the loading channel. When one cell was aspirated into the constriction channel, other cells loaded in the loading channel could be flushed to the bypass channel due to the hydraulic pressure between the inlet and bypass outlet. To ensure the cell movement inside the channels without restraint, the heights of the loading, bypass and release channels were 25 μm, and the maximum widths of the loading, bypass and release channels were 1000, 300 and 300 μm. As for the constriction channel, both the width and the height were designed to be 10 μm, which was ~60% of the average diameter of the A549 cells (~15–18 μm) for cell compression with good sealing and little cell disruption. A trapezoid inlet of the constriction channel (width: from 20 to 10 μm, length: 25 μm) was designed for the smooth entering of the aspirated cell. The total length of the constriction channel was 130 μm to make sure there was adequate time for cell electroporation during cell movement inside. 

The fabrication of the double-layer device was modified from previous publications (see Supplementary Figure S2) [43,44]. The first layer of Si to form the constriction channel was etched on a Si substrate (Supplementary Figure S2a,b). The second layer of SU-8 to form the cell loading, bypass and release channels of SU-8 photoresist was spin-coated on the Si substrate covering the first layer of Si, soft-baked, exposed with alignment (Supplementary Figure S2c), post-baked and developed (Supplementary Figure S2d). Next, polydimethylsiloxane (PDMS) prepolymer and curing agents were mixed in a ratio of 10: 1, degassed, poured on the double-layer Si + SU-8 mold and baked in an oven (Supplementary Figure S2e). Then, the PDMS was peeled from the mold, and punched for the inlet, bypass outlet and outlet (Supplementary Figure S2f). After oxygen plasma treatment, the PDMS was bonded to a glass substrate (Supplementary Figure S2g).

### 2.4. Single-Cell Electroporation and Impedance Measurement Protocol

In operations, firstly, propidium iodide (PI, Invitrogen, Carlsbad, CA, USA) with a concentration of 60 μg/mL was introduced to the cell suspension [25,45]. Meanwhile, the culture medium containing 60 μg/mL PI was pumped into the microfluidic channel to exhaust the air bubbles and maintain the experimental PI concentration. Afterwards, a droplet of cell suspension was pipetted into the inlet channel. A pair of Ag microelectrodes were put in the inlet and outlet, respectively. A bipolar square wave voltage with a low frequency of 1 kHz was applied to ensure adequate time for cell membrane charging [13] and also reduce the Joule heating [18]. By the negative pressure supplied from the pressure calibrator in the outlet, single cells could be aspirated to the constriction channel continuously. The negative pressure could be utilized to control the throughput of our microfluidic chip. For fluorescence observation, the corresponding negative pressure was set below 0.5 kPa to make sure single cells passing slowly in seconds. In order to improve the throughput, the pressure was improved to 1–2 kPa, which is an optimized pressure for single-cell electroporation and electrical detection based on our previous works [43]. The resulted passing time of most cells was around tens to hundreds of milliseconds. Simultaneously, the electrical signals were amplified by the trans-impedance amplifier (TIA) and recorded by the data acquisition (DAQ) card. The obtained raw data was the square-wave voltage on the feedback resistance of 100 kΩ, which could be directly derived into the circuit current. The envelope of the square-wave current was obtained utilizing Matlab. Then, the channel impedance variation could be calculated by dividing the applied voltage by the current envelope.

### 2.5. Cell Viability Test

To test the cell viability after electroporation, the electroporation assays without PI delivery were performed. After electroporation assays, the cells were cultured for 1 hour in a cell incubator at 37 °C in 5% CO_2_ for membrane recovery. Afterward, cells were stained with 2 μM Calcein-AM (Invitrogen, Carlsbad, CA, USA ) and 1 μg/mL PI and incubated for 15 min in the dark [26,45,46]. Then, both bright field and fluorescence images were captured by the fluorescence microscope to observe cell viability.

## 3. Results and Discussion

### 3.1. Theoretical Analysis of Single-Cell Electroporation Inside a Microchannel

Exposure of a cell to a sufficient electric field can induce cell transmembrane voltage (TMV) far exceeding its resting range and lead to electroporation of the membrane for intracellular delivery [13,42]. The occurrence of electroporation expresses like a quasi-threshold behavior, which is correlated with the TMV. Moreover, the critical TMV sufficient for electroporation was experimentally estimated to be hundreds to 1000 mV [35]. Therefore, efficient charging of cell TMV is demanding for electroporation applications. A constriction channel has a unique advantage of capturing a single cell inside. When the voltage was applied across the constriction channel, a high electric field would be localized on the captured cell, thus inducing a markedly high TMV. For a clear illustration, numerical simulations of cell TMVs during cell passing were deployed, as shown in Figure 2. With a low voltage of 3 V applied on the two ends of the microchannel (Figure 2a), the TMVs when the cell was outside (i), entering (ii) and fully elongated inside (iii) the constriction channel respectively, were simulated, showing significant differences. When the cell was loaded, the TMV was quite low, with the maximum value of 0.0138 V (Figure 2b(i)). However, when the cell was entering the constriction channel, the TMV was rapidly increased to hundreds mV on the two exposed membrane sections (Figure 2b(ii)). Furthermore, as the cell was entirely entered (Figure 2b(iii)), the TMV could reach a very high value of 1.2658 V, almost 100 times larger than that in the loading channel. The simulation results indicated that the cell entering the constriction channel was accompanied by the efficient charging of TMV. Applying a low voltage of 3 V could make the TMV increase to a high value, which might be sufficient for electroporation. Once the TMV reached a critical value for electroporation during cell entry, the cell would be electroporated and become permeable for the foreign cargos. Therefore, the constriction channel could be considered as the “electroporation region” for every aspirated cell.

**Figure 3:**
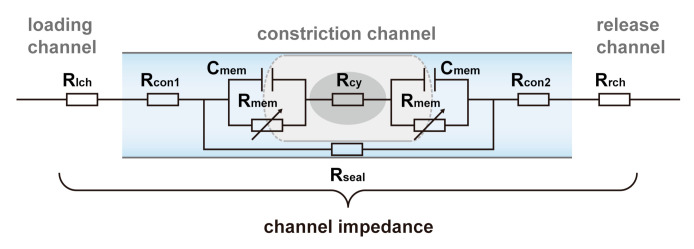
The equivalent electrical model of the single cell in the constriction channel.

For better understanding, an equivalent electrical model of the single cell in the constriction channel was developed (Figure 3). In this electrical circuit, the channel impedance consisted of the single-cell-occupied channel impedance and the resistances of the loading channel (R_lch_), the release channel (R_rch_) and the remained constriction channel (R_con1_ and R_con2_). The single-cell impedance included the impedance of two exposed cell membranes (containing C_mem_, R_mem_) and the cytoplasm resistance (R_cy_). Meanwhile, the sealing resistance (R_seal_) between the cell and constriction channel walls was introduced. As the cell was compressed in the constriction channel, the fluid between the cell and the contact walls was an extremely thin layer, resulting in the large sealing resistance. In addition, the cell membrane impedance was quite large before electroporation [44,47]. Therefore, the applied voltage would be mainly dropped on the single-cell-occupied channel impedance. As the resistance of cytoplasm is far less than the cell membrane impedance, the applied voltage would be mainly dropped on the exposed cell membranes (Figure 2b(iii)). Since the critical TMV for electroporation is around a few 100 mV, the constriction channel enables efficient single-cell electroporation with low applied voltage.

### 3.2. Single-Cell Electroporation Observation and Real-Time Impedance Monitoring

**Figure 4:**
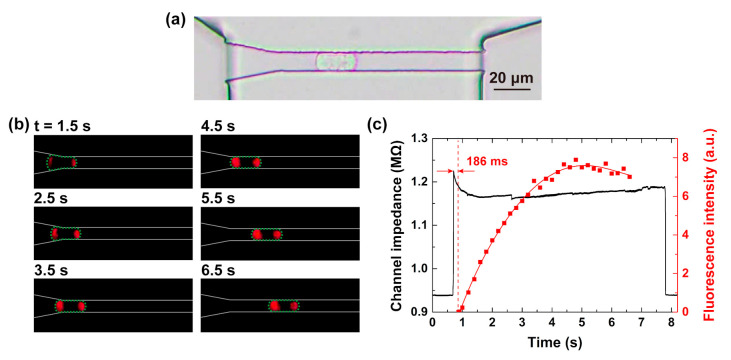
(**a**) Microscopic image of a single A549 cell inside a constriction channel observed in the bright field, (**b**) time-lapse fluorescent images of the propidium iodide (PI) delivery during cell passing with a voltage of 3 V. (**c**) The correspondences of the channel impedance (black line) and fluorescence intensity (red dot-line) over time when the cell in (**b**) passed through the channel.

In experiments, a microfluidic chip with a constriction channel was designed and fabricated. Electroporation of A549 cell lines and delivery of impermeable nucleic dye PI were demonstrated and evaluated. As shown in Figure 4a, a single cell was elongated in the constriction channel with proper sealing. Figure 4b displays the time-lapse fluorescent images of the PI delivery during cell passing with a voltage of 3 V applied on the two ends of the microchannel (Figure 1). Moreover, the correspondence of the fluorescent intensity of the cell over time was quantified (Figure 4c, red dot-line). Both from Figure 4b,c, one can observe that, when the cell was entering the constriction channel, the fluorescence intensity gradually increased in both sides of the cell, which demonstrated the occurrence of electroporation and the intracellular delivery of PI inside the constriction channel with a low voltage of 3 V [27].

Another advantage of our proposed microfluidic system is the capability to measure impedance variation in real-time when the single cell is electroporated inside the constriction channel, thus providing a label-free method to evaluate the electroporation efficiency. For this purpose, we simultaneously acquired the impedance variation induced by single-cell traveling and electroporation (Figure 4c, black line). The impedance above the baseline was derived from the entry and movement of the cell in the constriction channel. Interestingly, during cell entry, the channel impedance rapidly rose to a peak value and then abruptly dropped. By combining the impedance variation with the fluorescence intensity variation on the same time scale, we noticed that the impedance drop took place ~23 ms after the cell started entering the constriction channel, which was even ~186 ms earlier than the time when the fluorescence was detected for the first time. Based on the equivalent electrical model (Figure 3) and the observed fluorescence signals, we speculated that this abrupt impedance drop might be attributed to the occurrence of electroporation, which resulted in the decrease of the cell membrane resistance. Once the cell was electroporated, the cell status turned to a relatively stable level with impedance almost unchanged. Thus, “the door” was stably opened for the entry of foreign cargos. PI molecules could continuously cross the cell membrane, enter into the cell, bind to nucleic acids and generate fluorescence. It was observed that the fluorescence gradually increased for about 4 s before it reached the plateau region. This procedure for fluorescence accumulation was much slower than the detection of the abrupt impedance drop. The obtained results from the impedance and fluorescence intensity changes indicated that our proposed system could not only provide an efficient way for single-cell electroporation but also provide a sensitive measurement to monitor the electroporation procedure in real-time.

### 3.3. Electroporation Assessment Utilizing Channel Impedance Variation

**Figure 5:**
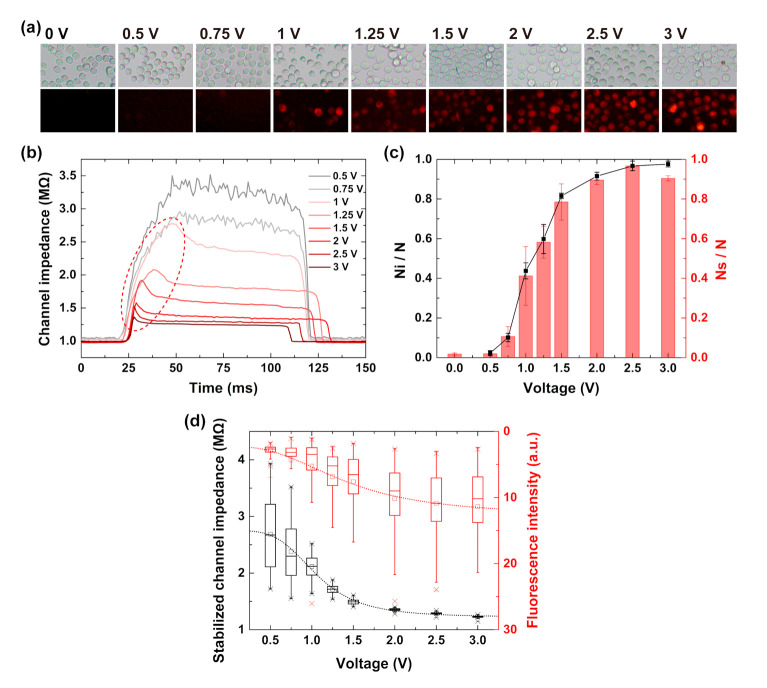
(**a**) Bright-field and fluorescent images of cells after experiments with voltage from 0 to 3 V. (**b**) The correspondences of the channel impedance over time when single cells passed through the channel with voltage from 0.5 to 3 V. (**c**) The ratios of the stained cells (Ns) (red column) and the cells with impedance drop (Ni) (black dot-line) to the total cells (N) at different voltages. (**d**) The trends of stabilized channel impedance (black box) and fluorescence intensity (red box) of single cells versus voltage.

The effects of the external voltage on the electroporation efficiency were also studied. Square waves with voltage from 0 to 3 V were applied on the chip, respectively, and the corresponding fluorescence intensity of PI delivery and channel impedance were both recorded and compared, as shown in Figure 3. For throughput improvement, the passing time of most cells were decreased to tens or hundreds of milliseconds by supplying the negative pressure of 1–2 kPa. We collected the total amount of the cells in the outlet and analyzed the fluorescent intensity for simplicity. When the cells passed through the constriction channel without voltage applied, no visible fluorescence signal was observed, which indicated that the mechanical deformation of the cell in the constriction channel was safe for the integrity of the cell membrane. Also, there was no visible fluorescence signal when the voltage was 0.5 V and only slight fluorescence when the voltage was 0.75 V, which implied that the electric fields were not strong enough to penetrate the membrane of most cells. There are several cells that had the visible fluorescence signal when the voltage was increased to 1 V. Furthermore, the amount of the cells with the fluorescent signal increased as the applied voltage increased from 1 to 3 V. Statistically, the electroporation efficiencies, which were defined as the ratios of the stained cells (Ns) to the total cells (N) at different voltages, were calculated and plotted in Figure 5c (red column). It was demonstrated that our microfluidic system could achieve a high electroporation efficiency above 90% with a relatively low voltage of 2 V. In addition, the electroporation efficiency reached 96.6% ± 0.2% when the applied voltage was 2.5 V.

Compared to the fluorescence observation of the collected cells at the outlet, the real-time impedance monitoring could reveal more insights into the electroporation effect at single-cell resolution with a high sensitivity. Figure 5b shows the channel impedance during single-cell-passing through the constriction channel at different voltages. When the voltage was lower than 0.75 V, no channel impedance drop was observed. When the voltage was larger than 1 V, the abrupt impedance drops started appearing after the impedance peaks (Figure 5b, red dash-line circle area), which showed similar curve features like that in Figure 4c. Similarly, we also calculated the ratios of the cells with impedance drop (Ni) to the total cells (N) at different voltages (Figure 5c, black dot-line). The highly consistent variation trends between Ni/N and Ns/N demonstrated our speculation that the appearance of the channel impedance drop during cell passing marked the occurrence of electroporation. From Figure 3, one could expect that the cell filling corresponded to the channel impedance increase, while the abrupt channel impedance drop was attributed to the rapid decrease of the cell membrane impedance. The constriction channel has the advantage of the gradual charging of the cell membrane during cell entry. Thus, the electroporation moment could be recorded in real time. Better than the “end-point” observation of the fluorescence intensity, the impedance drop detection using the constriction channel provides a label-free, real-time strategy for single-cell electroporation assessment.

Besides, the stabilized channel impedance when the cell was in the constriction channel could be used to reflect the electroporation extent under specific voltage. The obtained stabilized impedances were extracted when the single cells were in the center of the straight part of the constriction channel, as plotted in Figure 5d (black box). One can see that the stabilized channel impedance monotonously decreased with the increased voltage, indicating the enhanced extent of electroporation. Also, the diversity of impedances from cell to cell was gradually reduced when the voltage increased from 0.5 to 2 V. When the voltage was larger than 2 V, the impedance diversity among cells was minimal, meaning most cells were uniformly electroporated utilizing the constriction channel.

Furthermore, the corresponding fluorescence intensity of every single cell in the outlet was also measured (Figure 5d, red box). Similar to the features of the stabilized channel impedance, the fluorescence intensity was firstly slightly increased with voltage from 0.5 to 0.75 V, then rapidly increased with voltage from 1 to 1.5 V, and lastly, remained almost stable when the voltage further increased from 2 to 3 V. It was observed that the trend of fluorescence intensity increase was entirely consistent with the trend of stabilized channel impedance decrease, which confirmed the effectiveness of stabilized channel impedance as “physical marker” for cell electroporation assessment.

**Figure 6:**
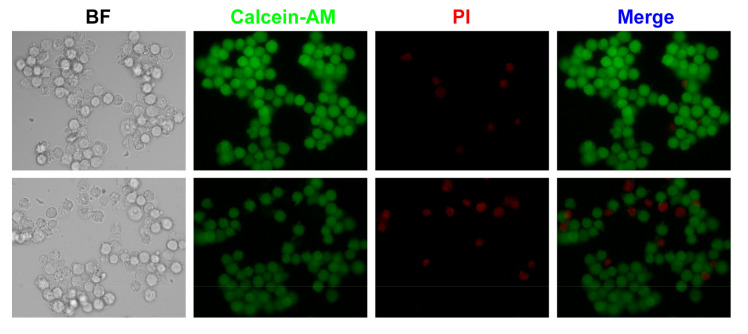
Cell viability test after electroporation with a voltage of 3 V.

In addition, cell viability is also an important parameter for effective electroporation. Based on the obtained results, one could see that the electroporation efficiency tended to be saturated when the applied voltage was above 2 V. To confirm whether the cell membrane permeabilization was reversible under these electric field conditions, we performed cell viability tests when the voltage was 3 V. In these tests, cells were traversing through the microfluidic channel without PI introduced. Afterwards, cells were incubated for 1 hour for cell membrane recovery. Then, a cell viability dye, Calcein-AM as well as PI were introduced to observe cell viability. As shown in Figure 6, one could find that cells maintained high viability (showing green fluorescence), and cell membrane of most cells were recovered as only a few cells were stained by PI (showing red fluorescence). Thus, with low applied voltage, this microfluidic system could achieve single-cell electroporation with high efficiency and high viability, suitable for the intracellular delivery applications.

## 4. Conclusions

In order to apply a localized high electrical field to every single cell, a constriction microchannel was introduced to make the applied voltage mainly loaded on the membranes of the traversing cells, since the resistance of the cell sealing is larger than the rest of the channel. Meanwhile, as a “physical maker”, the impedance variation during cell passing for electroporation effect assessment was studied. Referred by the fluorescence tracer, two marked features, including the abrupt impedance drop when cell entering, and the amplitude of stabilized impedance after the impedance drop, were found to indicate the occurrence of electroporation and meter the electroporation intensity, respectively. As a label-free maker, the impedance variation is possible for electroporation assessment during the intracellular delivery of cargos without a fluorescence tracer. Benefiting from both of the functions, a high electroporation efficiency of 96.6% in a continuous flow was achieved under a voltage of 2.5 V, which was much lower than the voltages (typically tens to hundreds) employed in the reported micro/nano-electroporation (MEP/NEP) systems. The results verified the potential of this microfluidic system for precise single-cell electroporation in intracellular delivery applications.

## Figures and Tables

**Table 1:** Simulation parameters.

The relative permittivity of the extracellular medium	80
The conductivity of the extracellular medium (S/m)	1.2
The relative permittivity of cytoplasm	80
The conductivity of cytoplasm (S/m)	0.4
The relative permittivity of membrane	34
The conductivity of the membrane (S/m)	10^−6^
The thickness of the membrane (nm)	10

## Supplementary Materials

The following are available online at https://www.mdpi.com/2072-666X/11/9/856/s1, Figure S1: Photograph of the microfluidic chip and the micrographs of the loading channel, bypass channel, constriction channel and release channel. Figure S2: The microfluidic device was fabricated based on the key steps of (a, b) deep etch of Si, (c) SU-8 25 spin coating, exposure with alignment, (d) development, (e) PDMS molding and (f) peeled PDMS with holes punched. After plasma treatment, the PDMS layer and the glass substrate were bonded together (g).
