# Supplementary material for: Single-Cell Electroporation with Real-Time Impedance Assessment Using a Constriction Microchannel
Source: Micromachines (Basel). 2020 Sep 16;11(9):856. doi: 10.3390/mi11090856 (PMC7570009; doi:10.3390/mi11090856)
Supplement: Supplementary file 1 [file micromachines-11-00856-s001.pdf]

micromachines

Supplementary Materials: Single-cell Electroporation with Real-time Impedance Assessment USING a Constriction Microchannel

Yifei Ye [1,2], Xiaofeng Luan [1,2], Lingqian Zhang [1], Wenjie Zhao [1,2], Jie Cheng [1,2], Mingxiao Li [1], Yang Zhao [1,*] and Chengjun Huang [1,2,*]

[1] R&D Center of Healthcare Electronics, Institute of Microelectronics, Chinese Academy of Sciences, Beijing,100029, China; yeyifei@ime.ac.cn (Y.Y.); luanxiaofeng@ime.ac.cn (X.L.); zhanglingqian@ime.ac.cn (L.Z.); zhaowenjie@ime.ac.cn (W.Z.); chengjie@ime.ac.cn (J.C.); limingxiao@ime.ac.cn (M.L.)

[2] School of Future Technology, University of Chinese Academy of Sciences, Beijing,100049, China

* Correspondence: zhaoyang@ime.ac.cn (Y.Z.); huangchengjun@ime.ac.cn (C.H.); Tel.: +86-010-8299-5600 (Y.Z.); +86-010-8299-5743 (C.H.)

Received: 17 August 2020; Accepted: date; Published: date

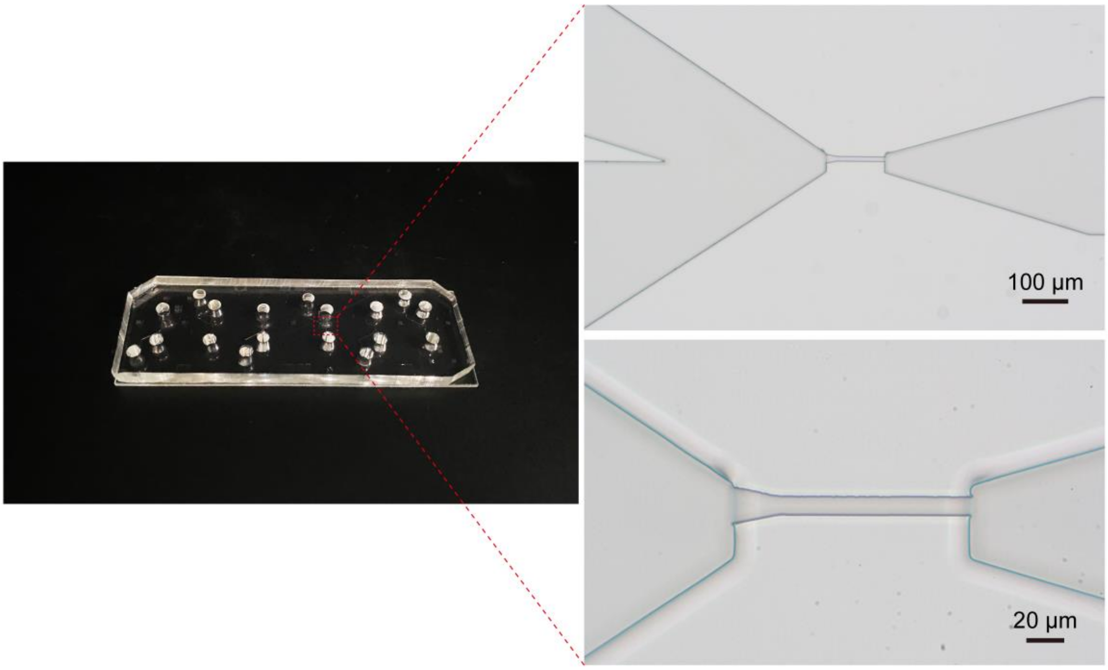

Figure S1. Photograph of the microfluidic chip and the micrographs of the loading channel, bypass channel, constriction channel and release channel.

.

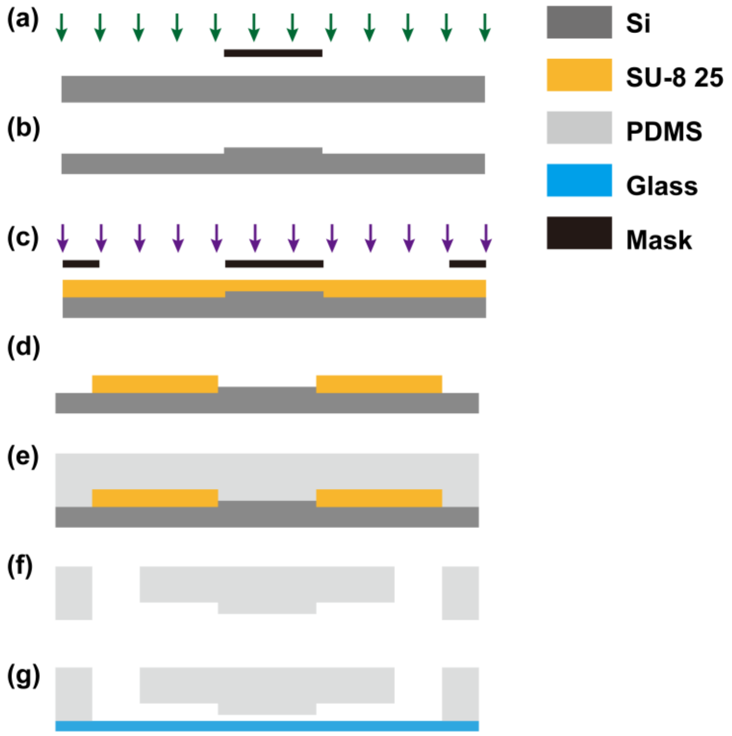

Figure S2. The microfluidic device was fabricated based on the key steps of (**a**,**b**) deep etch of Si, (**c**) SU-8 25 spin coating, exposure with alignment, (**d**) development, (**e**) PDMS molding and (**f**) peeled PDMS with holes punched. After plasma treatment, the PDMS layer and the glass substrate were bonded together (**g**).
